# Antitumor Effect of the Mannich Base(1,3-*bis*-((3-Hydroxynaphthalen-2-yl)phenylmethyl)urea) on Hepatocellular Carcinoma

**DOI:** 10.3390/molecules21050632

**Published:** 2016-05-14

**Authors:** Vadanasundari Vedarethinam, Karthik Dhanaraj, Soundharrajan Ilavenil, Mariadhas Valan Arasu, Ki Choon Choi, Naif Abdullah Al-Dhabi, Srigopalram Srisesharam, Kyung Dong Lee, Da Hye Kim, Tamilvenvendan Dhanapal, Ravikumar Sivanesan, Han Sung Choi, Young Ock Kim

**Affiliations:** 1 Department of Biotechnology, PRIST University, Thanjavur, Tamilnadu 613-403, India; vedavadana@gmail.com (V.V.); kar07bio@gmail.com (K.D.); 2 Grassland and Forage Division, National Institute of Animal Science, Chungnam 330-808, Korea; arulvenil@rediffmail.com (S.I.); choiwh@Korea.kr (K.C.C.); srigopal.ram@gmail.com (S.S.); 3 Department of Botany and Microbiology, Addiriyah Chair for Environmental Studies, College of Science, King Saud University, Riyadh 11451, Saudi Arabia; mvalanarasu@gmail.com (M.V.A.); naldhabi@ksu.edu.sa (N.A.A.-D.); 4 Department of Oriental Medicine Materials, Dongshin University, Naju 520-714, Korea; leek-d@hanmail.net; 5 United Graduate School of Agricultural Sciences, Tottori University, Tottori 680-8550, Japan; pioioiq@nate.com; 6 Department of Chemistry, National Institute of Technology, Tiruchirappalli 620-015, India; haitamilvendan@gmail.com; 7 Department of Emergency Medicine, College of Medicine, Kyung Hee University, Seoul 02447, Korea; 8 Development of Ginseng and Medical Plants Research Institute, Rural Administration, Eumseong 369-873, Korea

**Keywords:** 1,3-BPMU, HEP-G2 cells, DEN, apoptosis, ultrastructure

## Abstract

The present study was designed to evaluate the antitumor effects of the synthetic Mannich base 1,3-*bis*-((3-hydroxynaphthalen-2-yl)phenylmethyl)urea (1,3-BPMU) against HEP-G2 hepatoma cells and diethylnitrosamine (DEN)-induced hepatocarcinoma (HCC) in albino rats. *In vitro* analysis results revealed that 1,3-BPMU showed significant cytotoxicity and cell growth inhibition in HEP-G2 hepatoma cells in a concentration-dependent manner. Furthermore, flow cytometry results indicated that 1,3-BPMU enhanced early and late apoptosis. The maximum apoptosis was exhibited at a concentration of 100 μg/mL of 1,3-BPMU. In *in vivo* analysis, DEN treatment increased the content of nucleic acids, LPO and the activities of AST, ALT, ALP, LDH, γGT and 5’NT with decreased antioxidant activity as compared to control rats. However, 1,3-BPMU treatment to DEN-induced rats decreased the content of nucleic acids, LPO and the activities of AST, ALT, ALP, LDH, γGT and 5’NT and increased the activities of SOD, CAT, GPx, GST and GR (*p* < 0.05). Furthermore, 1,3-BPMU enhanced the apoptosis via upregulation of caspase-3 and caspase-9 and the downregulation of Bcl-2 and Bcl-XL mRNA expression as compared to DEN-induced rats. Histological and ultrastructural investigation showed that 1,3-BPMU treatment renovated the internal architecture of the liver in DEN-induced rats. In this study, the molecular and pre-clinical results obtained by treatment of DEN-induced rats with 1,3-BPMU suggested that 1,3-BPMU might be considered as an antitumor compound in the future.

## 1. Introduction

Hepatocellular carcinoma (HCC) is a primary liver cancer, the sixth most common malignant neoplasm, and 85% of HCC patients die in developing countries [1,2]. Risk factors for HCC include hepatitis B virus (HBV), hepatitis C virus (HCV), aflatoxins, alcohol and oral contraceptives [3], smoking, androgenic steroids and diabetes mellitus [4]. To trigger malignancy in different sites of the liver, numerous chemical carcinogen responses, which lead to varied patterns of cellular proliferation, occur in the development of HCC [5]. Carcinogenic substances are found in tobacco, meat, cosmetics, gasoline, many processed food items and alcoholic beverages [6]. Diethylnitrosamine, also known as N-nitrosodiethylamine (DEN), is extensively used as a carcinogen in the animal model. DEN is absorbed and metabolized by the pericentral zone and gets activated in the liver lobules. More activation leads to stimulated oxidative stress-mediated hepatocarcinogenesis through DNA damage by free radical formation, downregulated apoptotic genes and proteins, upregulated anti-apoptotic gene expression and protein synthesis [7].

Drug development and management are key techniques to develop new therapeutics to contest with HCC and to prolong patients’ life. Synthesizing novel drugs for liver cancer by combinational organic synthesis is still challenging, and there is a keen interest in accelerating drug discovery [8,9]. Blocking the carcinogenic pathway and mechanism of HCC, novel drugs have been developed and discovered from artificial and natural sources [10,11]. Many such sources were identified as antioxidants; for example, galangin is one of the flavonoids from plants that stimulates apoptosis via the mitochondrial pathway and could be considered as a potential antitumor compound against HCC [12]. 2′-Fluoro-6,7-methylenedioxy-2-phenyl-4-quinolone is a synthetic 6,7-substituted 2-phenyl-4-quinolone used as a potent antitumor agent for human HCC [13].

Mannich bases possess potent antiviral, antibacterial, antifungal and antioxidant activity in *in vitro* models [14]. The chemoresistance of tumor cells is considered as the primary reason for chemotherapeutic treatment failure. The reasons for drug resistance in tumor cells includes low intracellular drug concentration, changes in drug target interaction and changes in the cellular response. Therefore, we need to identify novel compounds for the treatment of tumor cells, especially high drug-target interaction compounds. For that purpose, we synthesize Mannich base-derived lead compounds and analyzed their interaction with a sialoglyco protein (a protein increased in HCC) using computational biology tools [15]. According to our previous experiments, we planned to investigate the pre-clinical and molecular approaches for a newly-synthesized Mannich base-derived organic compound, namely 1,3-*bis*-((3-hydroxynaphthalen-2-yl)phenylmethyl)urea (1,3-BPMU) in HEP-G2 hepatoma cells and DEN-induced rats.

## 2. Results

### 2.1. Cytotoxicity and Growth Inhibition Assay

**Figure 1:**
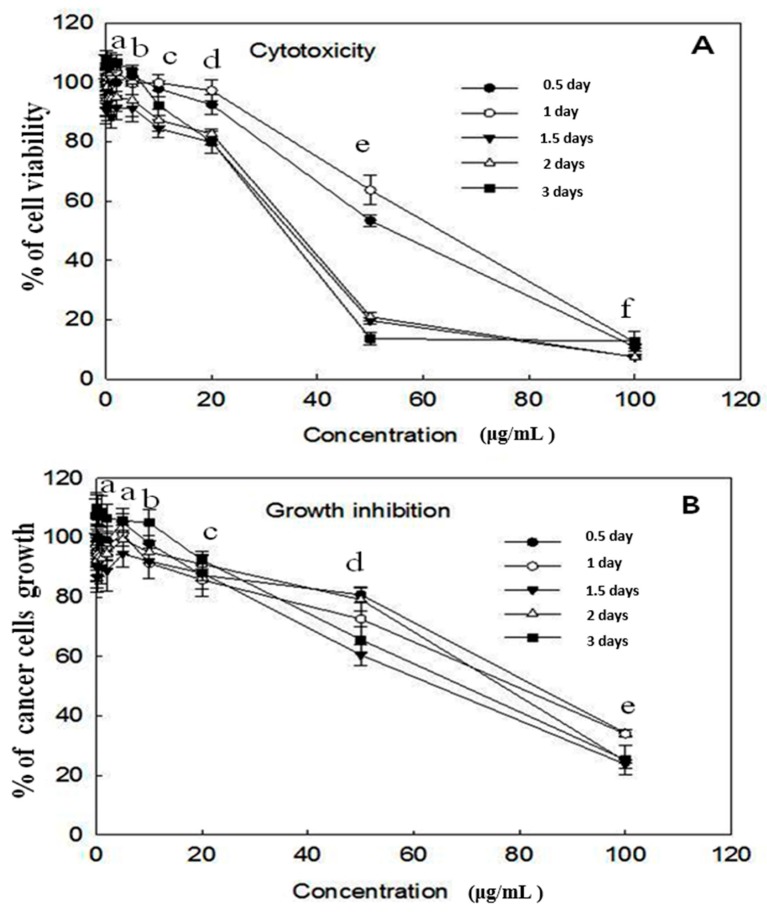
(**A**) Cytotoxicity effect of 1,3-BPMU on HEP-G2 hepatoma cells. The cells were treated with different concentrations of 1,3-BPMU in 10% FBS-DMEM and incubated for 0.5, 1.0, 1.5, 2.0 and 3 days at 37 °C. The maximum cytotoxicity was exhibited at the concentrations of 50 and 100 μg/mL of 1,3-BPMU; (**B**) Effect of 1,3-BPMU on HEP-G2 cell growth. The cells were treated with different concentrations of 1,3-BPMU in serum-free DMEM and incubated for 0.5, 1.0, 1.5, 2.0 and 3 days at 37 °C. 1,3-BPMU exhibited a potent tumor growth inhibition at 100 μg/mL. Different letters, a, b, c, d, e, f, within a treatment between concentrations of 1,3-BPMU indicate significant differences (*p* < 0.05).

HEP-G2 hepatoma cells were incubated with different concentrations of 1,3-BPMU (0.001, 0.01, 0.1, 0.2. 0.5, 1, 2, 5, 10, 20, 50 and 100 μg/mL) for 0.5, 1, 1.5, 2 and 3 days at 37 °C (*p* < 0.05). The results showed that 1,3-BPMU treatment significantly reduced the HEP-G2 cell viability in a time- and dose-dependent manner. Maximum cytotoxic effects were noted at 100 μg/mL of 1,3-BPMU (Figure 1A). Further, 1,3-BPMU treatment significantly inhibited the cell growth at concentrations of 50 and 100 μg/mL of 1,3-BPMU (Figure 1B).

### 2.2. Flow Cytometry Analysis

**Figure 2:**
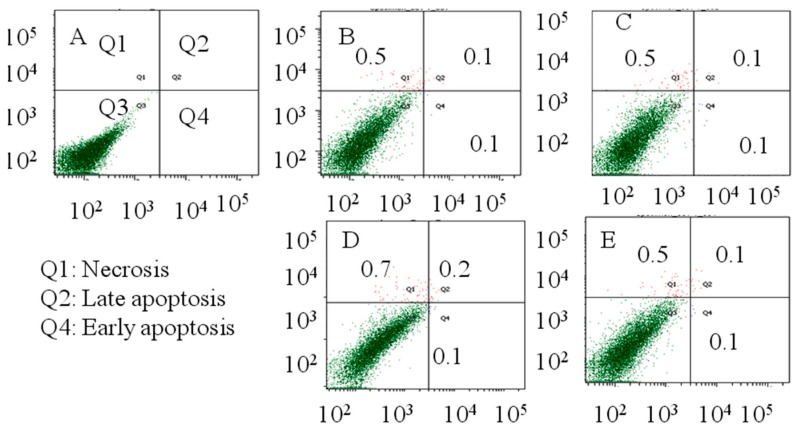

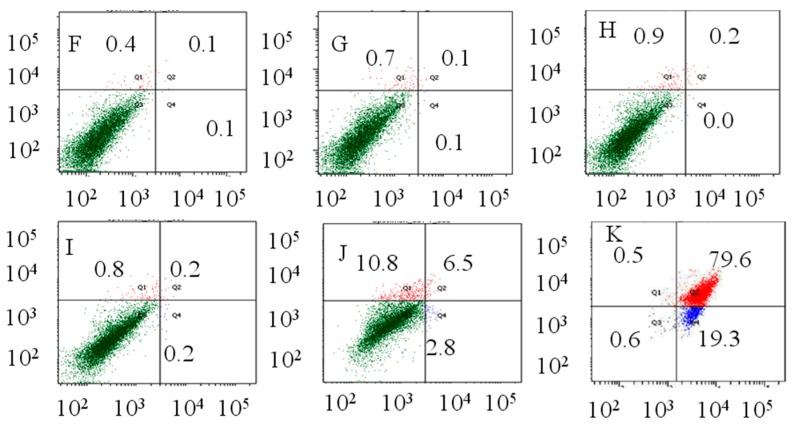
Apoptosis and necrosis quantification. (**A**) Control; (**B**) 0.1; (**C**) 0.2; (**D**) 0.5; (**E**) 1.0; (**F**) 2.0; (**G**) 5.0; (**H**) 10; (**I**) 20; (**J**) 50; (**K**) 100 μg/mL of 1,3-BPMU.

Figure 2A–K shows the percentage of apoptosis and necrosis in experimental cells. The results clearly indicated that 1,3-BPMU treatment significantly increased cellular necrosis, late apoptosis and early apoptosis in HEP-G2 cells in a dose-dependent manner. The maximum necrosis (0.5%), late apoptosis (79.6%) and early apoptosis (19.3%) were noted at the concentration of 100 μg/mL (*p* < 0.05).

### 2.3. Growth Conditions of the Rats

The normal rats excreted normal urine and gained significant amounts of body weight with healthy activity and good hair. Tumor-bearing rats exhibited poor health conditions, including lack of activity, loss of hair, lack of feed intake and slow growth. The body and liver weights of experimental rats are given in Table 1. The final body weight of tumor-bearing rats was significantly lower than the normal rats (*p* < 0.05). The 1,3-BPMU treatment of cancer-induced rats increased their body weight as compared to tumor control rats (*p* < 0.05) However, rats treated with 1,3-BPMU showed significantly reduced liver weight as compared to tumor-bearing rats. There were no significant alterations observed in rats treated with 1,3-BPMU alone as compared to control rats (*p* < 0.05).

### 2.4. Inhibitory Effect of 1,3-BPMU on Tumor Growth in DEN-Induced Rats

Numerous visible nodules were found in tumor-bearing rats and 1,3-BPMU with DEN-induced rats. Tumor-bearing rats had more large- and small-sized nodules (58.65 ± 1.02 nodules (>2 mm size) and 6.3 ± 0.05 nodules (>4mm)). 1,3-BPMU treatment of the cancer-bearing rats significantly reduced the number of nodules as compared to tumor-bearing rats (*p* < 0.05). The mean volume of nodules in the 1,3-BPMU treatment of cancer-bearing rats was less than in tumor-bearing rats (Table 2).

### 2.5. Estimation of DNA and RNA

Table 3 represents the quantification of DNA and RNA levels in the experimental groups of rats. The levels of DNA and RNA were increased in tumor-bearing rats as compared to normal rats, whereas cancer-bearing rats treated with 1,3-BPMU exhibit reduced levels of DNA and RNA as compared to tumor control rats. The elevation of DNA levels is associated with malignancy development. This indicates the cell proliferation activity under tumor conditions. There were no significant changes observed in rats treated with 1,3-BPMU alone as compared to normal rats.

### 2.6. Estimation of Tumor Markers (AFP and CEA) Concentration

Table 4 shows tumor marker proteins, such as α-fetoprotein (AFP) and carcinoembryonic antigen (CEA), in the experimental groups of rats. The levels of AFP and CEA cancer markers were elevated in tumor rats as compared to normal control (*p* < 0.05). However, 1,3-BPMU treatment significantly reduced the tumor marker levels in tumor rats as compared to tumor control rats (*p* < 0.05). There were no significant variations observed in rats treated with 1,3-BPMU alone as compared to normal rats.

### 2.7. Activities of Serum Marker Enzymes, Lipid Peroxidation and Antioxidant Status

Table 5 shows the AST, ALT and ALP, LDH, γGT and 5’NT activities in experimental rats. Tumor control rats showed increased activities of AST, ALT, ALP, LDH, γGT and 5’NT as compared to normal rats, whereas, cancer-bearing rats treated with 1,3-BPMU showed a significant reduction in AST, ALT, ALP, LDH, γGT and 5’NT activities as compared to tumor rats (*p* < 0.05). Rats treated with 1,3-BPMU alone did not exhibit significant changes in AST, ALT, ALP, LDH, γGT and 5’NT activities as compared to normal rats.

The effects of 1,3-BPMU on lipid peroxide (LPO) and antioxidant status in the experimental group of rats are shown in Table 6. The LPO level was increased in tumor control animals as compared to normal rats (*p* < 0.05). DEN-induced rats administered with 1,3-BPMU showed a significant reduction of LPO levels. The antioxidant enzymes, such as SOD, CAT, GPx GR and GST, were reduced in tumor-bearing rats (*p* < 0.05). However, cancer-induced rats treated with 1,3-BPMU had reversed antioxidant status as compared to tumor-bearing rats (*p* < 0.05). No significant changes were noted in rats treated with 1,3-BPMU alone as compared to normal rats.

### 2.8. Apoptotic and Anti-Apoptotic Gene Expression Analysis by qPCR

**Figure 3:**
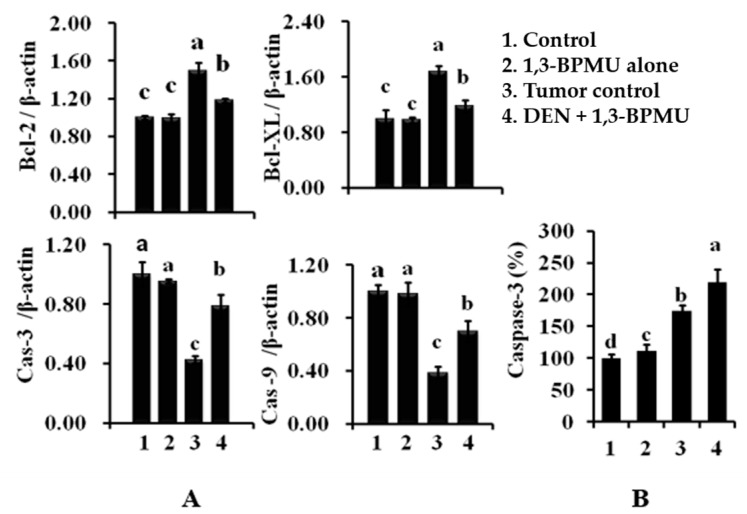
Impact of 1,3-BPMU on apoptotic and anti-apoptotic gene expression in control and experimental animals. (**A**) Bcl-2, Bcl-XL, caspase-3 and caspase 9 mRNA expression in experimental rats; (**B**) Quantification of caspse-3 protein expression in experimental rats. Different letters, a, b, c, d, within a treatment between concentrations of 1,3-BPMU indicate significant differences (*p* < 0.05).

Figure 3A shows apoptotic and anti-apoptotic gene expression in the liver of experimental animals. The expression of apoptotic genes, such as caspase-3 and caspase-9, was decreased in tumor-bearing rats as compared to the normal control rats, whereas DEN-induced rats treated with 1,3-BPMU displayed significantly increased caspase-3 and caspase-9 expression as compared to the tumor-bearing rats. The anti-apoptotic genes, such as Bcl-2 and Bcl-XL, increased their expression in tumor-bearing rats as compared to normal rats, whereas tumor-bearing rats treated with 1,3-BPMU had downregulated Bcl-2 and Bcl-XL expressions as compared to tumor-bearing rats. Figure 3B shows colorimetric quantification of caspse-3 expressions in experimental rats. The caspase-3 protein level was decreased in tumor-bearing rats as compared to normal rats, whereas 1,3-BPMU treatment of DEN-induced rats showed increased capase-3 expression as compared to tumor-bearing rats.

### 2.9. Histology and Ultrastructural Study

**Figure 4:**
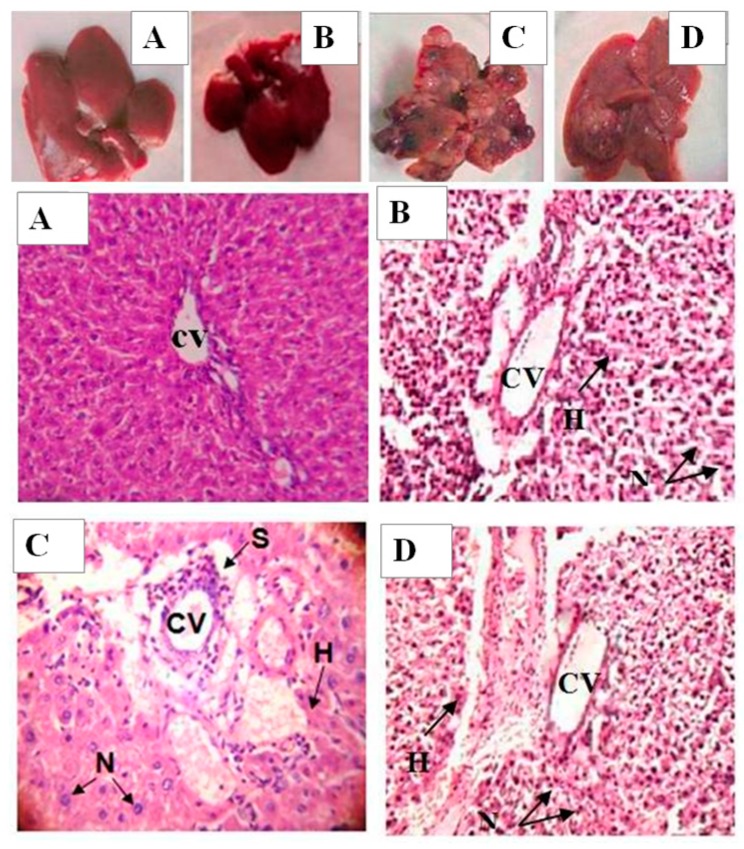
Histological analysis of liver tissue by hematoxylin-eosin staining. (**A**) Normal rats; (**B**) 1,3-BPMU alone; (**C**) tumor control; (**D**) DEN + 1,3-BPMU rats. Sinusoids (S), nucleus (N), central vein (CV) and hepatocyte (H).

Figure 4 shows histological observations of liver sections of experimental rats. The numbers of visible nodules were higher in the tumor-bearing rats than 1,3-BPMU-treated rats. The normal rats exhibited normal architecture, central vein, normal size of hepatocyte with the proper nucleus and granulated cytoplasm. The tumor-bearing rats exhibited an unformatted architecture, enlarged nuclear size and more inflammatory cells around the central vein. The cancer-bearing rats treated with 1,3-BPMU significantly reverted to the normal structure of liver as compared to tumor-bearing rats.

**Figure 5:**
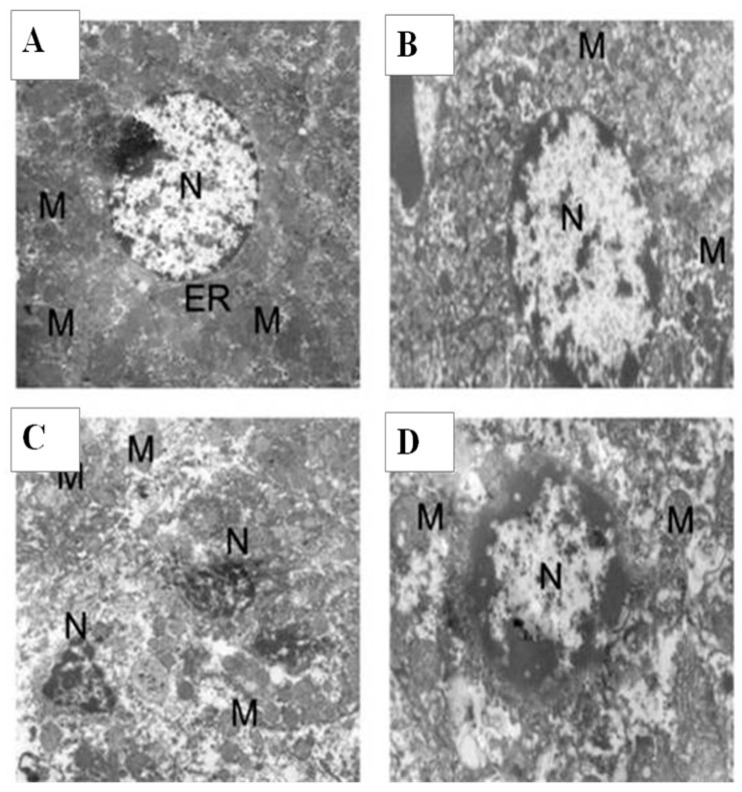
Ultrastructural analysis of liver tissue by transmission electron microscope (TEM). (**A**) Normal rats; (**B**) 1,3-BPMU alone; (**C**) tumor control; (**D**) DEN + 1,3-BPMU rats. Mitochondria (M), endoplasmic reticulum (ER) and nucleus (N).

The ultra-structural changes that occurred in hepatocytes of experimental rats are shown in Figure 5. Electron micrographs of the hepatocytes of normal rats showed intact cellular organelles, mitochondria, a nucleus with a nuclear membrane and a rough endoplasmic reticulum. A similar architecture was observed in rats that were treated with 1,3-BPMU alone. Tumor-bearing rats showed damaged intracellular organelles, with an unshaped nucleus and improper cytoplasm and mitochondria arrangement, as well as numerous mitochondria of abnormal size. The cancer rats treated with 1,3-BPMU showed significantly restored nuclear and mitochondrial size and shape. This indicates that 1,3-BPMU has the ability to prevent cancer induced by DEN.

## 3. Discussion

The liver plays a pivotal role in the regulation of physiological processes. Due to oxidative stress, macromolecules, such as lipids, proteins and other molecules, get disturbed. In this study, initially, we evaluated the cytotoxicity and growth inhibition ability of 1,3-BPMU in HEP-G2 hepatoma cells. The activation of cell cycle control proteins is a major mechanism for many DNA-damaging agents, including irradiation, and most antitumor compounds used in the clinic that stimulate DNA damage [16]. The maximum cytotoxicity effects were observed at a concentration of 100 μg/mL on the third day. Further, 1,3-BPMU significantly inhibits the cell growth at 50 and 100 μg/mL by the third day of incubation. The cell cycle progress is an important indicator of the molecular mechanism of cell death and cell cycle progression [17]. Flow cytometry analysis is used to find out the apoptotic alteration in the cells [18]. The apoptotic effect of 1,3-BPMU was examined by flow cytometer (FITC-annexin-V), and the results revealed that maximum (0.5%), late apoptosis (79.6%) and early apoptosis (19.3%) were noted at the concentration of 100 μg/mL of 1,3-BPMU. These results suggested that 1,3-BPMU extensively induced apoptosis in HEP-G2 hepatoma cells with an increase of incubation time.

Generally, carcinogens stimulate the formation of free radical and non-radical oxidizing species in animals and humans in pathologic environments [19]. DNA acts as a genetic determinant and functional aspect of all tumor formations. In our present study, DNA and RNA synthesis were elevated in experimental groups because nucleic acids are involved in multiple functions, such as cell division and cell differentiation, *etc*. Uncontrolled cell division and cell differentiation lead to tumor progress. We noted increased DNA and RNA levels in cancer-bearing rats, which indicates that the DEN treatment increased the replication and transcription process in rats. However, 1,3-BPMU treatment of cancer-bearing animals reduced nucleic acid biosynthesis; this indicated that 1,3-BPMU can control cancer cell growth through inhibition of nucleic acids synthesis. Furthermore, 1,3-BPMU treatment of the cancer-bearing rats showed reduced numbers of nodules and tumor volume. This result strongly correlated with nucleic acid level, body and liver weight of rats in the 1,3-BPMU-treated group.

The carcinoembryonic antigen (CEA) is one of the immunoglobulin supergene families used to detect tumors by its functions, such as adhesion, forming homotypic and heterotypic aggregates between the cells [20]. α-Fetoprotein is another oncofetal glycoprotein. It is not found in normal healthy people. It is the best marker for the detection of HCC. AFP along with CEA are widely used in the diagnosis of HCC [21,22,23]. In the present study, high levels of AFP and CEA were noted in the DEN-induced rats. This confirmed the development of HCC in rats. However, treatment of cancer-bearing rats with 1,3-BPMU significantly decreased the level of AFP and CEA as compared to tumor rats.

Administration of 1,3-BPMU to the DEN-induced rats improved the metabolic function of the liver by decreasing the liver marker level in cancer-bearing rats. The activities of AST and ALT will increase with the incidence of hepatic diseases and abnormal metabolic function. AST is an enzyme found in the liver, heart, skeletal muscles, kidneys and, to a lesser extent, in the pancreas [24]. An increase of these markers is usually associated with cardiac arrest or liver disease. Furthermore, ALP, LDH and γGT activities were increased in cancer-bearing rats, which indicated the abnormal metabolism of hepatic cells. ALT and AST increases lead to the development of hepatocellular damage. In our study, we noted increased levels of ALT and AST in cancer-bearing rats. This is great evidence for cancer development in rats caused by the DEN treatment. However, cancer-bearing rats treated with 1,3-BPMU significantly normalized the elevated levels of ALT and AST. The leakage of LDH indicates non-specific changes in plasma membrane integrity. Liberation of γGT present in the plasma membrane into serum indicates liver cell damage. In the present study, cancer-bearing rats showed higher levels of LDH and γGT in the blood, which indicates that DEN disturbed the integrity of the plasma membrane. The 1,3-BPMU treatment of cancer-bearing rats significantly reduced the level of these enzymes as compared to tumor rats, confirming that 1,3-BPMU maintains the membrane integrity of the liver. Liver marker enzyme 5’NT is present on the surface of the plasma membrane of hepatocytes and acts as a tool for the diagnosis of liver injury [25,26,27]. Elevated levels of the 5’NT marker enzyme in tumor rats are correlated with malignancy development. 1,3-BPMU treatment decreased the activity of 5’NT as compared to tumor rats.

Lipid peroxides play an important role in carcinogenesis and cellular injury. In general, DEN administration generates LPO, like MDA, that may stimulate oxidative stress and carcinogenesis. The highest level of LPO is noted during cancerous conditions in the rats. The activity of LPO leads to the uncompromised production of free radicals that overcome the cellular antioxidant defenses [28,29,30,31]. The present study exhibited increased levels of LPO in tumor rats as compared to normal rats. However, 1,3-BPMU-treated rats showed reduced levels of LPO as compared to tumor control rats. This indicates 1,3-BPMU controls the free radical generation in the cancer-bearing rats.

Antioxidant enzymes play a major role in protecting the cell from various compounds, like reactive oxygen species (ROS) and chemical carcinogens. SOD, CAT, GPx, GST and GR play the main role in the elimination of ROS [32,33]. Superoxide dismutase is involved in the scavenging of superoxide radicals, and it acts as the first line of defense against superoxide phosphorylation. In the present study, decreased activities of antioxidant enzymes, such as SOD, CAT, GPx, GST and GR, were observed in cancer-bearing rats, which indicates that DEN-induced oxidative stress leads to HCC. The 1,3-BPMU treatment of cancer-bearing rats significantly improves the antioxidant status as compared to untreated cancer-bearing rats. The results indicated that administration of 1,3-BPMU helps to restore the hepatic cellular function through a free radical scavenging mechanism.

Caspases are groups of aspartate-specific cysteine proteases that regulate the apoptosis induced by different kinds of stimuli. Caspase-3 is an effector gene involved in the apoptotic process, and caspase-9 acts as an initiator of caspase-3 in the mitochondria-dependent pathway. Generally, the caspases regulate apoptosis through DNA fragmentation, chromatin condensation and nuclear fragmentation. Caspase-3 activation could be caused by upstream proteases, either caspase-3 in the intrinsic pathway or caspase-8 in the extrinsic pathway in the death receptors [34]. In the present study, cancer-bearing rats showed reduced expression of caspse-3 and caspase-9 as compared to normal control rats. This indicates that DEN injection blocks the apoptosis program through downregulation of caspase-3 and caspse-9, but cancer-bearing rats administered with 1,3-BPMU show significantly increased caspase-3 and caspse-9 expression. This upregulation of these genes enhances the apoptotic process. Bcl-2 genes are important regulators for cytochrome C release from mitochondria and caspases activation. These groups contain both apoptotic and antiapoptotic genes. Bcl-XL expressions prevent mitochondrial cytochrome C release, protecting the cells from apoptosis by inhibiting the availability of cytochrome C in the cytosol [35]. In our study, DEN treatments upregulated the Bcl-2 and Bcl-XL mRNA transcripts in liver of rats as compared to normal rats. Results confirmed that DEN treatment induced cell proliferation and inhibits cell apoptosis in rats. Increased cell proliferation and inhibition of cell apoptosis cause tumor and cancer, whereas in cancer-bearing rats treated with 1,3-BPMU, Bcl-2 and Bcl-XL mRNA transcripts were significantly downregulated as compared to cancer-bearing rats. From these results, it is concluded that 1,3-BPMU significantly activates the apoptotic process through upregulation of apoptotic genes and downregulation of anti-apoptotic genes. Previously, we analyzed the effect of 1,3-BPMU on protein expression changes in DEN-induced rats. That confirmed that 1,3-BPMU regulates numerous proteins associated with cancer prevention mechanisms in rats [36].

Histopathological and transmission electron microscopic investigations were performed to evidence morphological changes in experimental rats. H & E-stained normal liver tissue showed normal architecture arrangement and the normal size of hepatocytes with the nucleus and granulated cytoplasm. The cancer-bearing rats exhibited an unformatted architecture, enlarged nuclear size and more inflammatory cells around the central vein. The cancer-bearing rats treated with 1,3-BPMU significantly reversed to a normal liver structure as compared to cancer rats. TEM results showed that cancer-bearing rats exhibited irregularly-shaped nucleus and cytoplasm; metastatic stages were seen, which might be due to the excessive free radical generation. Normal and 1,3-BPMU alone-treated rats exhibited a similar kind of architecture; cancer-bearing rats treated with 1,3-BPMU show signs of the stimulation of apoptosis, like shrunken nucleus, condensed chromatin membrane and the formation of apoptotic bodies. This indicates that administration of 1,3-BPMU restores the hepatic cellular architecture and hepatic cellular function through the apoptotic mechanism. All of the biochemical results significantly correlated with the histological studies.

## 4. Materials and Methods

### 4.1. Chemicals

Diethylnitrosamine (DEN) and phenobarbital were obtained from Sigma Aldrich (St Louis, MO, USA), and the remaining chemicals used in this study were of analytical grade. Human HEP-G2 hepatoma cells (ATCC-HB-8065) were procured from the American Type Culture Collection (ATCC, Manassas, VA, USA).

### 4.2. Synthesis of Mannich Base

**Scheme 1:**
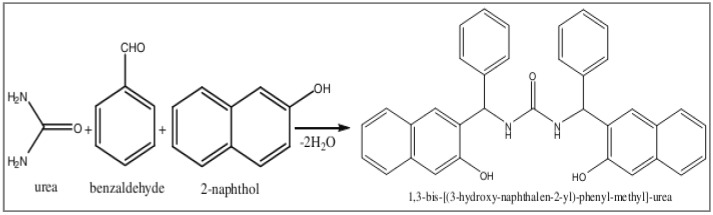
Reaction scheme for 1,3-*bis*-[(3-hydroxynaphthalen-2-yl) phenylmethyl]urea.

Formulation and characterization of 1,3-BPMU were carried out by Dr. Tamilvendan Dhanapal at the National Institute of Technology (Trichy, India) according to Scheme 1 [14].

### 4.3. Cytotoxicity and Growth Inhibition Assay

Cell toxicity was assessed by the MTT assay. Briefly, HEP-G2 hepatoma cells were maintained in DMEM culture medium supplemented with 10% FBS at 5% CO_2_ at 37 °C and sub-cultured in 96 wells containing serum-free media (SFM) at a density of 3 × 10^4^ cells/well, exposed to different concentrations of 1,3-BPMU (0.001, 0.01, 0.1, 0. 2 0.5, 1, 2, 5, 10, 20, 50 and 100 μg/mL) for 0.5, 1, 1.5, 2 and 3 days at 37 °C. Then, the cells were treated with MTT reagent and placed into a 5% CO_2_ incubator at 37 °C for 4 h [37]. The growth inhibitory effect of 1,3-BPMU was studied as follows: 5 × 10^3^ HEP-G2 hepatoma cells seeded in 96-well plates with 100-μL volumes of growth media. Then, the cells were treated with different concentrations of 1,3-BPMU in DMEM media supplemented with 10% serum. The absorbance was measured at 560 nm.

### 4.4. Flow Cytometric Analysis of Apoptosis

FITC-annexin V (sc-4252 FITC, Santa Cruz biotechnology, Santa Cruz, CA, USA) was used for analyzing the apoptosis, and propidium iodide (PI) reagent (P4170, Sigma) was used for identifying the necrotic cells. Cells were treated with different concentrations of 1,3-BPMU (0.001, 0.1, 0. 2, 0.5, 1, 2, 5, 10, 20, 50 and 100 μg/mL) for 48 h, and then, experimental cells were harvested and resuspended in binding buffer (10 mM HEPES pH 7.4, 150 mM NaCl, 5 mM KCl, 1 mM MgCl_2_ and 1.8 mM CaCl_2_) containing FITC-annexin V (1 μg/mL) and incubated for 20 min. About 10 minutes before the end of incubation, PI (10 μg/mL) was added to this cell suspension in order to stain necrotic cells. These cells were analyzed with a FACS flow cytometer (Becton Dickenson Biosciences, San Jose, CA, USA) equipped with an excitation laser line at 488 nm. The PI was collected through a 575 ± 15-nm bandpass filter [38].

### 4.5. Animals

Male 150–170 g Wister albino rats (*Rattus norvegicus*) were selected for the experimental study. All rats were housed in polypropylene cages at 25 ± 2 °C with 40%–55% relative humidity in dark and light conditions (each 12 h). The rats were fed with normal feed and water *ad libitum.* The Institutional Animal Ethics Committee (743/03/abc/CPCSEA dt: 03.03.2003) and Committee for the Purpose of Control and Supervision of Experiments on Animals (CPCSEA, Chennai, India) approved the experiments.

### 4.6. Experimental Design

The rats were segregated into four groups each consisting of six animals. The experimental periods were 16 weeks as follows:

Group A: Normal rats fed with standard diet with *ad libitum* access to water.
Group B: Rats received 1,3-BPMU alone (50 mg/kg/bw/day/orally based on the IC_50_) at 24-h intervals.
Group C: Tumor control; hepatocarcinoma was induced by a single dose intraperitoneal (ip) injection of DEN (200 mg/kg/bw), and further, rats were administered with phenobarbital (250 mg/kg/bw/day/orally for two weeks) as a cancer-promoting agent [39].
Group D: 1,3-BPMU-treated; hepatocarcinoma was induced by a single dose intraperitoneal (ip) injection of DEN (200 mg/kg/bw); then, rats were administered with phenobarbital (250 mg/kg/bw/day/orally) for two weeks as a cancer-promoting agent; then, animals were treated with 1,3-BPMU (50 mg/kg/bw/day/orally) up to the end of the experiment periods.

At the end of the experimental periods, the body weight was recorded, and then, rats were anesthetized with sodium pentothal. All rats were sacrificed by cervical dislocation. Liver samples were collected and weighed. A small portion of the liver was homogenized in 0.1 M Tris buffer, pH 7.4 and used for further assays. The visible size of the tumor was measured using a Vernier caliper, and the volume of the largest nodules was calculated [40].

### 4.7. Analysis of Biochemical Parameters

Tumor markers, such as α-fetoprotein (AFP) and carcinoembryonic antigen (CEA), were quantified using a chemiluminescent immunoassay method. The activities of aspartate transaminase (AST), alanine transaminase (ALT), alkaline phosphatase (ALP), lactate dehydrogenase (LDH), superoxide dismutase (SOD), catalase (CAT), glutathione peroxidase (GPx), glutathione-*S*-transferase (GST), glutathione reductase (GR) γ-glutamyltransferase (γ-GT), 5’-nucleotidase and levels of lipid peroxide (LPO), DNA and RNA were estimated [41,42,43].

### 4.8. Expression of Genetic Analysis by qPCR (Caspse-3, Caspase-9, Bcl-2 and Bcl-XL)

The RNA was extracted from experimental liver tissues using a commercial kit (RNA lipid tissue mini kit, Qiagen, Venlo, Netherlands) according to the manufacturer’s protocol. The extracted RNA was estimated using a UVS-99 Micro-volume UV-VIS spectrophotometer (ACTGene, Piscataway, NJ, USA). One microgram of RNA was used to synthesize cDNA using oligo (dT) nucleotide and III reverse (superscript III first stand synthesis system-cDNA synthesis kit-Applied biosystems, Kookmin, Korea). The qPCR was conducted with an ABI 7500 Real-Time PCR System (Applied biosystems). Target gene expressions were determined by SYBR green-based real-time PCR in 20-µL reactions containing 10 µL Power SYBR Green Master Mix (Applied Bio-Systems, Foster City, CA, USA), 1 µL cDNA, 1 µL 10 pmole forward (FP) and reverse primers (RP). qPCR cycling protocol; polymerase activation 95 °C, 3 min; denaturation 95 °C, 15 s, annealing 58 °C, 30 s; Melting curve analysis 55–95 °C with 0.5 °C increment. caspase-3-FP: ATG TCG ATG CAG CTA ACC TC; RP: TCC TTT TGC TGT GAT CTT CC; caspase-9 FP: TCC TGC TTA GAG GAC ACA GG; RP: TGC TCC TTT GAT TTG AGT CC; Bcl-2 FP: GAC TCA CTA TAG GCG GGA GAT CGT G; RP: CAC TAT AGA GAA GGG CGT CAG GTG C; Bcl-XL FP: GAG CCA GAT CAT GTT TGA AGC CTT; RP: GGT GAC CGT AAC ACT ACC TGA G. β-actin FP: GACCCAGATCATGTTTAGGACCTT; RP: GGTGACCGTAACACTACCTGAG. The gene expression was normalized against the β-actin transcript signal.

### 4.9. Caspase-3 Quantification

The caspase-3 protein expression in experimental tissues was quantified by a commercial kit (ApoTarget, Invitrogen, Kookmin, Korea). Briefly, the tissues were resuspended in 50 µL of cell lysis buffer and incubated on ice for 10 min and then centrifuged at 10,000× *g* for a min. The supernatant was collected; the protein concentration in each sample was estimated; an equal concentration of protein from each sample was taken; and 50 µL of 2× reaction buffer and 5 µL of 4 mM DEVD-pNA substrate were added. Further, this was incubated at 37 °C for 2 h. Then, the absorbance of samples was measured at 400 nm using a microplate reader. The percentage of caspase-3 expression was calculated from caspase-2 in the normal control. Caspase-3 in normal control cells was considered as 100%.

### 4.10. Histology and Ultra-Structural Investigation

Two to three pieces of liver tissue, approximately 5 mm in size and fixed in 10% formalin solution and embedded in paraffin wax, were stained with hematoxylin-eosin [44,45]. The stained tissues were examined under the high-resolution microscope. For TEM analysis, the tissues were embedded in a mixture of (1:1) 1,2-epoxypropane and Epon (Epikote resin) and hardened using dodecyl succinic anhydride (DDSA) and methylnadic anhydride (MNA). Ultrathin sections were cut and stained with uranyl acetate and lead nitrate and collected on mesh grids coated with a thin Formvar film and viewed in an EM201C transmission electron microscope (Philips, Amsterdam, The Netherlands).

### 4.11. Statistical Analysis

Each experiment was carried out in replicates (*n* = 6). Data are expressed as the mean and the standard error of the mean (SEM). Statistical analysis was carried out using Excel (Microsoft, Washington, WA, USA) and one-way ANOVAs and multivariate comparisons using the Statistical Package for Social Science (SPSS) program Version 16.0 (SPSS, Inc., Chicago, IL, USA); significance was represented as *p* < 0.05.

## 5. Conclusions

The Mannich base 1,3-BPMU exhibited significant cytotoxicity and cell growth inhibition in HEP-G2 hepatoma cells. In addition, it stimulated apoptosis in HEP-G2 hepatoma cells. Further, it reduced the nucleic acids, lipid peroxide, cancer marker proteins and the activities of pathophysiological liver markers and increased the antioxidant activities in DEN-induced rats, and 1,3-BPMU also stimulates apoptosis via upregulation of caspase-3 and caspase-9 with downregulation of Bcl-2 and Bcl-XL gene expression. Histological and ultrastructural studies showed that 1,3-BPMU maintained the architecture of the liver in DEN-induced rats. This investigation suggested that 1,3-BPMU might be considered as a potent antitumor compound in the future. Further experiments are still needed to validate the molecular mechanism of action of 1,3-BPMU.

## Figures and Tables

**Table 1:** Body and liver weight of control and experimental rats.

Body Weight (g)	Control	1,3-BPMU Alone	Tumor Control	DEN + 1,3-BPMU *
Initial	156 ± 10.21 ^a^	162 ±12.8 ^a^	157 ± 12.9 ^b^	159 ± 12.5 ^c^
Final	202 ± 11.20 ^a^	205 ± 9.20 ^a^	184 ± 11.8 ^b^	208 ± 10.8 ^c^
Liver	7.45 ± 0.59 ^a^	8.20 ± 0.65 ^a^	13.29 ±1.2 ^b^	8.32 ± 0.67 ^c^

The results are expressed as the mean ± SD for six rats in each group. ^b^ *p* < 0.05 as compared to normal control and 1,3-BPMU alone rats ^a^; ^c^ *p* < 0.05 as compared to tumor control rats. * 1,3-BPMU 50 mg/kg/bw/day/orally and DEN 200 mg/kg/bw/ip.

**Table 2:** Effect of 1,3-BPMU on hepatocarcinoma incidence.

Groups	Average Number of Nodules in Liver		Volume of Nodules (mm^3^)
	(>2 mm)	(>4 mm)	
Normal control	0 ^a^	0 ^a^	0 ^a^
1,3-BPMU alone	0 ^a^	0 ^a^	0 ^a^
Tumor control	58.65 ± 1.02 ^b^	6.3 ± 0.05 ^b^	192.55 ± 2.85 ^b^
DEN + 1,3-BPMU *	34.21 ± 0.98 ^c^	2.6 ± 0.03 ^c^	72.25 ± 0.24 ^c^

The results are expressed as the mean ± SD for six rats in each group. ^b^ *p* < 0.05 as compared to normal control and 1,3-BPMU alone rats ^a^; ^c^ *p* < 0.05 as compared to tumor control rats. * 1,3-BPMU 50 mg/kg/bw/day/orally and DEN 200 mg/kg/bw/ip.

**Table 3:** Effect of 1,3-BPMU on the levels of nucleic acids in livers of control and experimental groups of rats.

Nucleic Acids (mg/g Wet Tissue)	Normal Control	1,3-BPMU Alone	Tumor Control	DEN + 1,3-BPMU *
DNA	1.8 ± 0.04 ^a^	5.72 ± 0.14 ^a^	8.10 ± 0.08 ^b^	2.28 ± 0.13 ^c^
RNA	1.3 ± 0.09 ^a^	2.70 ± 0.07 ^a^	5.3 ± 0.20 ^b^	1.58 ± 0.08 ^c^

The results are expressed as the mean ± SD for six rats in each group. ^b^ *p* < 0.05 as compared to normal control and 1,3-BPMU alone rats ^a^; ^c^ *p* < 0.05 as compared to tumor control rats. * 1,3-BPMU 50 mg/kg/bw/day/orally and DEN 200 mg/kg/bw/ip.

**Table 4:** Effects of 1,3-BPMU on cancer marker proteins α-fetoprotein and carcinoembryonic antigen in control and experimental groups of rats.

Groups	AFP (ng/mL)	CEA (ng/mL)
Normal control	0.05 ± 0.003 ^a^	0.04 ± 0.003 ^a^
1,3-BPMU alone	0.05 ± 0.004 ^a^	0.03 ± 0.005 ^a^
Tumor control	1.58 ± 0.07 ^b^	2.47 ± 0.050 ^b^
DEN + 1,3-BPMU *	0.43 ± 0.05 ^c^	0.18 ± 0.090 ^c^

The results are expressed as the mean ± SD for six rats in each group. ^b^ *p* < 0.05 as compared to normal control and 1,3-BPMU alone rats ^a^; ^c^ *p* < 0.05 as compared to tumor control rats. * 1,3-BPMU 50 mg/kg/bw/day/orally and DEN 200 mg/kg/bw/ip.

**Table 5:** Effects of 1,3-BPMU on serum marker enzymes of control and experimental rat groups.

Groups	AST (U/L)	ALT (U/L)	ALP (U/L)	LDH (U/L)	γGT (U/L)	5’NT (U/L)
Normal control	44.0 ± 2.42 ^a^	27.08 ± 1.08 ^a^	33.17 ± 1.21 ^a^	5.9 ± 0.36 ^a^	6.09 ± 0.32 ^a^	3.08 ± 0.38 ^a^
1,3-BPMU alone	43.0 ± 1.63 ^a^	26.05 ± 1.11 ^a^	34.12 ± 1.12 ^a^	5.6 ± 0.35 ^a^	6.08 ± 0.33 ^a^	3.0 ± 0.38 ^a^
Tumor control	192.2 ± 2.03 ^b^	82.36 ± 1.67 ^b^	75.46 ± 1.39 ^b^	12.3 ± 0.30 ^b^	12.38 ± 1.05 ^b^	6.19 ± 0.78 ^b^
DEN + 1,3-BPMU *	98.2 ± 3.79 ^c^	36.04 ± 1.33 ^c^	34.23 ± 1.99 ^c^	8.1 ± 0.24 ^c^	5.13 ± 0.46 ^c^	4.38 ± 0.51 ^c^

The results are expressed as the mean ± SD for six rats in each group. ^b^ *p* < 0.05 as compared to normal control and 1,3-BPMU alone rats ^a^; ^c^ *p* < 0.05 as compared to tumor control rats. * 1,3-BPMU 50 mg/kg/bw/day/orally and DEN 200 mg/kg/bw/ip.

**Table 6:** Effect of 1,3-BPMU on lipid peroxidation and antioxidant status of control and experimental groups of rats.

Groups	LPO	SOD (U/mg)	CAT (U/mg)	GPx (U/mg)	GR (U/mg)	GST (U/mg)
Normal control	2.97 ± 0.11 ^a^	3.99 ± 0.19 ^a^	4.82 ± 0.23 ^a^	6.21 ± 0.43 ^a^	5.28 ± 0.26 ^a^	0.89 ± 0.02 ^a^
1,3-BPMU alone	2.42 ± 1.16 ^a^	4.35 ± 0.21 ^a^	4.61 ± 0.22 ^a^	6.72 ± 0.33 ^a^	5.63 ± 0.28 ^a^	0.96 ± 0.02 ^a^
Tumor control	3.96 ± 0.19 ^b^	2.56 ± 0.27 ^b^	3.88 ± 0.28 ^b^	2.63 ± 0.38 ^b^	3.82 ± 0.19 ^b^	0.59 ± 0.01 ^b^
DEN + 1,3-BPMU *	2.99 ± 0.14 ^c^	4.87 ± 0.24 ^c^	4.72 ± 0.24 ^c^	5.73 ± 0.33 ^c^	4.83 ± 0.24 ^c^	0.71 ± 0.02 ^c^

LPO U/mg of protein; activity is expressed as µmol of GSH oxidized per min per mg of protein for GPx; units per min per mg of protein for GST; 50% inhibition of epinephrine auto-oxidation for SOD; µmole of hydrogen peroxide decomposed per min per mg of protein for CAT and µmole of NADPH oxidized/(min/mg protein) for GR. The results are expressed as the mean ± SD for six rats in each group. ^b^ *p* < 0.05 as compared to normal control and 1,3-BPMU alone rats ^a^; ^c^ *p* < 0.05 as compared to tumor control rats. * 1,3-BPMU 50 mg/kg/bw/day/orally and DEN 200 mg/kg/bw/ip.

## Abbreviations

1,3-BPMU: 1,3-*bis*-[(3-hydroxy-naphthalen-2-yl)-phenyl-methyl]-urea
DEN: Diethyl nitrosamine
HCC: Hepatocarcinoma
AFP: α-fetoprotein
CEA: Carcinoembryonic antigen
AST: Aspartate transaminase
ALT: Alanine transaminase
ALP: Alkaline phosphatase
LDH: Lactate dehydrogenase
LPO: Lipid peroxide
SOD: Superoxide dismutase
CAT: Catalase
GPx: Glutathione peroxidase
GST: Glutathione-S-transferase
GR: Glutathione reductase
γ-GT: γ-glutamyl transferase
